# College and Hospital Notes

**Published:** 1903-09

**Authors:** 


### COLLEGE AND HOSPITAL NOTES.

The Medical Department of the University of Buffalo will begin its fifty-eighth annual session Monday, September 28, 1903. The advantages possessed by this renowned school of medicine are many and it is excelled by none in the practical training it gives its pupils. The cost of living in Buffalo is moderate, the college fees are reasonable and the location of the building is an admirable one in every sense of the term, while men and women are received on equal terms. These and other reasons should weigh with those who desire to enter upon a medical career. For details address Dr. John Parmenter, secretary of the faculty, 24 High street, Buffalo, N. Y.

---

The Buffalo Fresh Air Mission Hospital, located at Athol Springs, on the lake shore, a few miles from Buffalo, possesses many advantages for the treatment of children. It receives not only babies suffering from cholera infantum and summer diarrheas, but also children with rickets, tuberculosis, bone and glandular diseases. Physicians may avail themselves of the privileges of the hospital for their private patients. The hospital carriage meets every train.

---

THE Beahan Hospital at Canandaigua has been changed in name to the Canandaigua Physicians' and Surgeons' Hospital.

---

Two new hospitals will be built at Albany, one a smallpox hospital, the other a pavilion for the reception and care of patients suffering from contagious diseases. The city will loan its credit to sustain the former,—a wise provision that should be adopted in other cities.

